# Performance of Electrochemical Processes in the Treatment of Reverse Osmosis Concentrates of Sanitary Landfill Leachate

**DOI:** 10.3390/molecules24162905

**Published:** 2019-08-10

**Authors:** Annabel Fernandes, Oumaima Chamem, Maria José Pacheco, Lurdes Ciríaco, Moncef Zairi, Ana Lopes

**Affiliations:** 1 FibEnTech-UBI, Department of Chemistry, Universidade da Beira Interior, 6201-001 Covilhã, Portugal; 2 Water, Energy and Environment Laboratory, National School of Engineering of Sfax (ENIS), Sfax 3038, Tunisia

**Keywords:** reverse osmosis concentrate, sanitary landfill leachate, anodic oxidation, electro-Fenton, electrocoagulation

## Abstract

Electrochemical technologies have been broadly applied in wastewaters treatment, but few studies have focused on comparing the performance of the different electrochemical processes, especially when used to treat highly-polluted streams. The electrochemical treatment of a reverse osmosis concentrate of sanitary landfill leachate was performed by means of electrocoagulation (EC), anodic oxidation (AO) and electro-Fenton (EF) processes, and the use of different electrode materials and experimental conditions was assessed. All the studied processes and experimental conditions were effective in organic load removal. The results obtained showed that EC, with stainless steel electrodes, is the cheapest process, although it presents the disadvantage of sludge formation with high iron content. At high applied current intensity, AO presents the best treatment time/energy consumption ratio, especially if the samples’ initial pH is corrected to 3. However, pH correction from natural to 3 deeply decreases nitrogen-containing compounds’ removal. For longer treatment time, the EF process with a carbon-felt cathode and a BDD anode, performed at natural iron content and low applied current intensity, is the most favorable solution.

## 1. Introduction

In the last decades, electrochemical processes have been being developed and applied in wastewaters treatment, combined or not with other techniques, to solve environmental problems. Promising results were achieved when compared to other technologies, especially in the treatment of effluents containing high organic load and recalcitrant compounds [1,2,3,4,5,6,7,8,9].

Sanitary landfill leachates are among the effluents that may pose major environmental concerns worldwide and, in particular, the leachate concentrates that result from the leachates treatment by membrane processes [10,11]. These effluents, characterized by a complex composition that includes different heavy metals, organic and inorganic compounds, some of them refractory and toxic, are very difficult to depurate, and several studies have focused on their treatment by electrochemical processes [10,11,12,13,14,15,16,17,18,19].

The most studied electrochemical technologies for sanitary landfill leachate treatment are electrocoagulation (EC), anodic oxidation (AO) and electro-Fenton (EF), with their efficacy being dependent on the effluent properties and experimental conditions applied during the electrochemical treatment [20].

Electrocoagulation has demonstrated to be an effective and practical technology to treat leachates from sanitary landfills, since it allows the treatment of effluents containing dissolved and non-dissolved pollutants without chemicals addition [21,22,23]. There are several process variables that can influence the EC treatment efficiency [7,18,21,24]. According to the literature, EC efficacy is generally enhanced when operated at neutral pH conditions and without mechanical stirring [7,25]. Iron and aluminum are the most widely used electrode materials, leading to high treatment efficacies [7,15]. However, the passivation of aluminum electrodes, which is detrimental to the reactors’ performance, the high operating costs reported with this metal, and its high toxicity when compared to iron, makes the use of Fe electrodes preferable [7,15,24].

Anodic oxidation is the most popular electrochemical procedure for landfill leachates treatment, since it is a simple and clean method that does not produce sludge and does not require electrolyte addition, due to the high conductivity presented by these effluents [7]. The application of AO to landfill leachates treatment has been reported by numerous authors, with very promising results [7,10,12,14,17,25]. Among the main operational parameters than can influence this process are the anode material and the applied current density. Due to its extraordinary properties, such as inert surface and extremely high O_2_ evolution overvoltage, boron-doped diamond (BDD) anodes are the most used in AO and have been reported to yield the highest oxidation rate of organic compounds and the highest current efficiencies [1,7,9].

Electro-Fenton is another promising electrochemical process for the treatment of landfill leachates and its concentrates and, according to the literature, it yields good treatment efficiencies and performs the oxidation of the organic pollutants at lower energetic costs than AO, if the operating parameters are optimized [7,10,11,26]. In EF, both species, H_2_O_2_ and Fe^2+^, can be electrogenerated in situ. H_2_O_2_ can be generated in solution through O_2_ bubbling, provided that a suitable cathode, like carbon-felt, is used for the two-electron reduction of O_2_ [7]. Fe^2+^ ions are continuously electro-generated at the cathode from the reduction of the Fe^3+^ ions, hence creating a catalytic cycle which accelerates the mineralization process [7]. According to the literature [2,27,28], low initial iron concentrations (0.1-0.2 mM) are required to operate the EF process and, depending on the iron concentration present in the effluent, the addition of extra iron may not be necessary [11]. Ferrous ion concentrations between 60 and 80 mg L^−1^ are pointed out in the literature as optimal for leachates treatment by the EF process [29]. Due to the influence of pH on iron speciation and on H_2_O_2_ decomposition, the optimum pH for the EF process is 3 [3]. The anode material is another key factor in the EF process. BDD and Pt have been widely used as anodes the in EF process, since they present good chemical stability, even at high potentials and in very corrosive media, and their use can accelerate the degradation of organic matter through the hydroxyl radicals formed as an intermediate of the anodic water discharge [30,31]. Pt anode, with low oxygen evolution overpotential, favours the partial and selective oxidation of pollutants in wastewater treatment, whilst BDD, with high oxygen evolution overpotential, is ideal for the complete electrooxidation of organic carbons to CO_2_ [9].

Several studies have also been performed using consumable iron anodes, a process usually called peroxi-coagulation. In this process, pollutants removal is achieved by coupling H_2_O_2_ production with electrocoagulation, accomplished by the Fe(OH)_3_ precipitate formed due to the excess of ferrous ion, increasing the efficiency of the process [32].

Although EC and AO have been extensively studied for the treatment of landfill leachates, with the influence of the operational parameters well established, the application of EF to the degradation of this type of highly polluting effluents has not yet been fully explored, considering the different possible approaches. Furthermore, there are several studies reporting the application of electrochemical technologies in landfill leachates treatment, but few were focused on comparing the efficacy of these methods to eliminate organic pollutants. In this context, this work aims to study the efficiency of EF to treat a reverse osmosis concentrate (ROC) of a sanitary landfill leachate, investigating the effect of different operating parameters, namely initial dissolved iron concentration, anode material and applied current intensity, and compare it to other electrochemical technologies (EC and AO) regarding organic load and nitrogen removal, iron content in the treated samples, and energy requirements.

## 2. Results and Discussion

### 2.1. Initial Samples Characterization

According to the electrochemical process to be applied and the experimental conditions to be used, the ROC sample was previously prepared and characterized. Four different initial samples were used: natural ROC sample (for AO assays), ROC sample acidified at pH 3 (for EF and AO assays), ROC sample acidified at pH 7 (for EC assays) and ROC sample acidified at pH 3 with iron addition by anodic dissolution of iron electrodes (for EF assays). Samples characterization results, presented in Table 1, show that, by acidifying the sample, a slight decrease in the organic load and nitrogen contents occurred. Also, inorganic carbon concentration decreased due to the formation of gaseous CO_2_. Following the pH adjustment during the sample’s preparation, the sample’s color changed from dark to light brown, and the formation of a precipitate was observed. The decrease in organic load and nitrogen contents is probably due to the flocculation of some dissolved matter and to some pollutants’ oxidation. Since samples were acidified with sulfuric acid, sulfate concentration significantly increased in the acidified samples, achieving a concentration of approximately 15 g·L^−1^ in samples acidified at pH 3. Also, electric conductivity increased with the samples’ acidification.

### 2.2. Electrochemical Experiments

**Figure 1:**
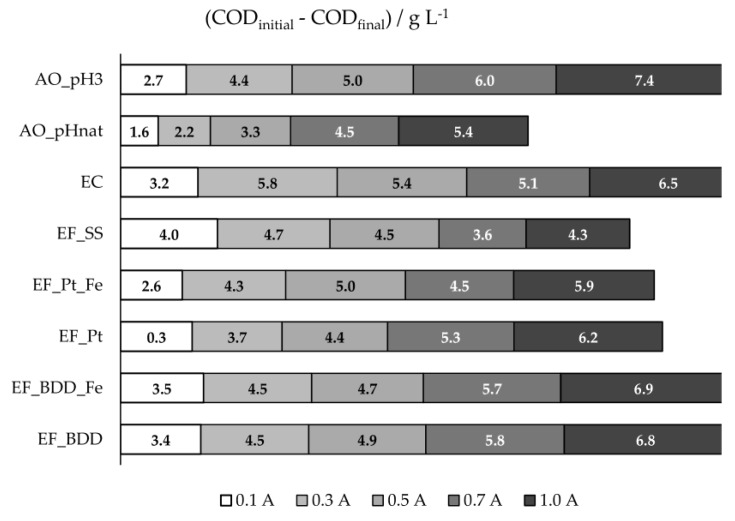
COD 8-h removals, in g L^−1^, for the assays performed at different experimental conditions.

Figure 1 and Figure S1 present the COD removals obtained at the different electrochemical experiments performed. For the EF assays performed with BDD or Pt anodes, it can be seen that, excluding the EF experiment performed with Pt and added iron (EF_Pt_Fe), COD removal increased with applied current intensity (I). This result can be explained by the increase in I that promotes higher formation of hydroxyl radicals, either by Fenton’s reaction or anodic water discharge, and sulfate and chloride oxidized active species that enhance the oxidation of the organic matter. The decrease in COD removal observed in EF_Pt_Fe when I was varied from 0.5 to 0.7 A can be due to side reactions that may occur (Equation (1)), reducing the oxidation rate of the organic matter in bulk solution. When I was further increased to 1.0 A, the amount of oxidizing species was so high that the side reactions became negligible, being the pollutants oxidation in the bulk solution enhanced, increasing COD removal.

HO^•^ + Fe^2+^ → Fe^3+^ + OH^−^ (1)

When EF experiments with Pt (EF_Pt) and BDD (EF_BDD) are compared, COD removals were higher when BDD anode was used. This can be due to the higher oxidation ability of BDD(^•^OH) when compared to Pt(^•^OH), reported in the literature [30,33]. The presence of extra iron did not influence the COD removal in EF_BDD. However, in EF_Pt, small differences were found, depending on the applied current intensity.

Comparing the results obtained in EF_BDD with those achieved in AO experiments, using the same anode material, it can be seen that, at the lowest applied current intensities, COD removal was higher for the EF process, confirming the important oxidation role of HO^•^ produced in the bulk solution by Fenton’s reaction. However, when I was increased, the AO performed with the acidified ROC sample (AO_pH3) showed to have better performance, since the formation of sulfate and chloride oxidized active species, which act in the bulk, was enhanced by the increase in applied current intensity. In fact, significant differences were found between AO experiments performed at natural (AO_pHnat) and acidified pH. AO_pH3 presented higher COD removals that can be explained by the decrease of CO_3_^2-^ and HCO_3_^−^ ions concentration when the effluent was acidified (DIC concentration decreased from 2.1 to 0.10 g·L^−1^ (Table 1)), which was advantageous, since these ions could scavenge hydroxyl radicals, decreasing the oxidation of the organic matter [34].

**Figure 2:**
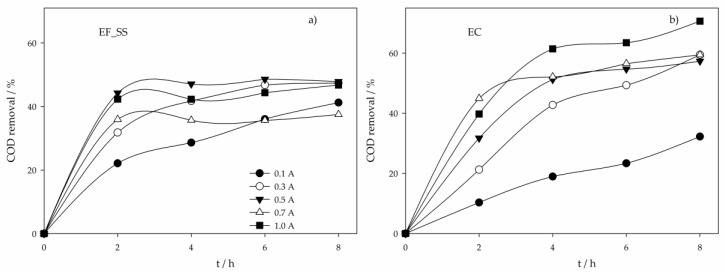
COD removal in time for the assays performed with a SS anode at various applied current intensities and using different electrochemical processes: (**a**) Electro-Fenton; (**b**) Electrocoagulation.

From all the electrochemical processes assayed, EF using SS as anode (EF_SS) presented the highest COD removal at the lowest applied current intensity, 0.1 A. This could be explained by the simultaneous occurrence of oxidation through HO^•^, produced by Fenton’s reaction, and coagulation and flotation, caused by the formation of coagulants by SS electrolytic oxidation. For higher I values, the solution pH gradually increased for values higher than 6 (Figure S2), achieving values above 9 at 0.7 and 1.0 A, increasingly hindering the Fenton’s reaction due to the absence of H_2_O_2_ production. In fact, it could be seen (Figure 2a) that the highest COD removal rates were accomplished in the first 2 hours of the assay, where the pH values were between 3 and 6.5. The fact that these removal rates, in the first 2 hours of the assay, were much higher than those obtained in EC (Figure 2b), especially at lower I, where the increase in pH in EF is not so pronounced, support the existence of pollutants’ oxidation by Fenton process. In EF_SS, when higher increases in pH were observed, electrocoagulation was the main oxidation process. However, stripping of the H_2_ formed was hindered by the cathode porous structure, thus reducing organic matter removal by flotation and explaining the higher COD removals observed for EC at higher I values, when compared to EF_SS.

**Figure 3:**
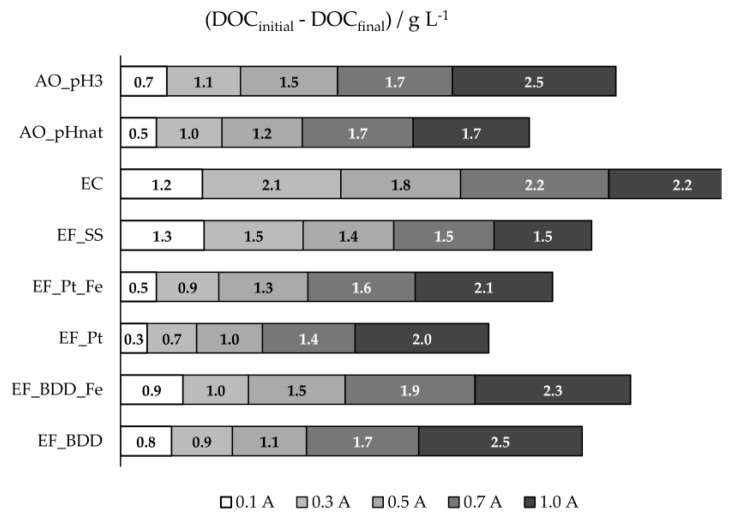
DOC 8-h removals, in g·L^−1^, for the assays performed at different experimental conditions.

DOC removal obtained in the different electrochemical processes utilized is presented in Figure 3 and Figure S3, for the different applied current intensities. Except for the processes that utilized stainless steel as anode (EF_SS and EC), DOC removal increased with applied current intensity, due to the increase in anodic oxidation. EF assays with BDD anode presented higher DOC removals than EF with Pt, probably due to the greater oxidation ability of BDD(^•^OH) compared to Pt(^•^OH), as described above. For these two systems, the addition of extra iron promoted an increase in DOC removal, except for EF_BDD performed at 1.0 A. As stated above, in the presence of extra iron, side reactions (Equation (1)) may occur with the increase in I, reducing the oxidation rate of the organic matter in bulk solution. At these conditions, more organic compounds will reach the anode surface, and since HO^•^ formation at the anode surface, from water discharge, is enhanced by the increase in I and the oxidation by these hydroxyl radicals favors the compounds’ mineralization instead of their partial oxidation, DOC removal will increase.

When EF_BDD is compared to AO_pH3, it can be observed that, only at the lowest applied current intensity, EF presented a better performance, once again showing the influence of the current intensity on the HO^•^ formation at the anode surface and its effect on the mineralization degree. The AO performed at natural pH presented lower DOC removal than at the initial pH of 3, which is similar to what happens for COD removal, due to the oxidation of the inorganic carbon, as referred to above. Regarding the assays performed with the SS anode, either EF or EC, DOC and COD removals, in percentage, were very similar, meaning that the organic matter removal was mainly due to precipitation/flotation of the electro-coagulated products. For these assays, COD and DOC removals tend to a plateau, meaning that the organic material with a tendency to flocculate and sediment is all removed during the first hours of the experiments.

Regarding nitrogen removal, results presented in Table 2 and Figure S4 show that the highest removals were attained for EC and AO_pHnat experiments. Nitrogen removal occurs mainly through indirect oxidation by active chlorine species, which is enhanced at neutral and alkaline pH conditions [35,36]. At acidic pH, the primary active chlorine species is Cl_2_, a strong oxidant that can suffer stripping, hindering its action as an oxidizing agent. Furthermore, at acidic pH conditions, Cl_2_ reaction with nitrogen leads to the formation of chloramines, which are very persistent to oxidation. At higher pH values, HClO and ClO^−^ are formed, these being the species unaffected by stripping and used as oxidizing reagents [36]. At 0.1 A, TN removals were low or inexistent for all the electrochemical experiments, since the current was not enough to produce active chlorine species. Between 0.3–0.7 A, the highest TN removals were achieved in the EC process, due to the optimal pH conditions for nitrogen removal through indirect oxidation by active chlorine species (as explained above) associated with the removal through coagulation and flotation. In fact, with the increase in pH and temperature, NH_4_^+^ is partially converted to NH_3_, which is stripped with gases formed around the cathode [13]. At 1.0 A, the TN removal decreased in the EC experiments, due to the sharp increase in pH that promoted the complete deprotonation of HClO to ClO^−^, a weaker oxidant.

The highest TN removal at 1.0 A was achieved by the AO_pHnat, since these are the most favorable conditions for active chlorine species production. In the EF assays performed with BDD, TN removal was higher in the experiments with extra iron at 0.1 to 0.5 A. At 0.7 and 1.0 A, the higher TN removals were achieved in the assays performed with natural iron conditions. In the experiments performed with extra iron (EF_BDD_Fe), since HO^•^ production is enhanced by the increase in iron concentration, some nitrogen oxidation could occur through hydroxyl radicals. However, with the increase in I, Cl_2_ production is enhanced and the formation of chloramines may reduce TN removal. The same explanation is valid for TN removals in EF with Pt—in this process the competition between organic carbon and nitrogen oxidations is more pronounced—since COD removal is not as effective as when BDD anode is used.

By using electrochemical processes mediated by iron, a concern about the amount of iron present in the treated effluent is raised. Total dissolved iron concentration was monitored along all the electrochemical experiments; the values attained after the 8-h assays are presented in Table 3.

For AO experiments, a decrease in iron concentration was found due to its reduction in the cathode, with the corresponding deposition on its surface [25]. For the EF assays with BDD, an increase in dissolved iron concentration was found, probably due to the dissolution of iron precipitates present in the ROC sample. On the other hand, for the EF assays with Pt, iron concentration slightly changed when assays were performed with natural iron concentration, but decreased when extra iron was added. This behavior can be explained by the iron deposition on the cathode, which increases with iron concentration, and was more pronounced when Pt was used [25].

**Figure 4:**
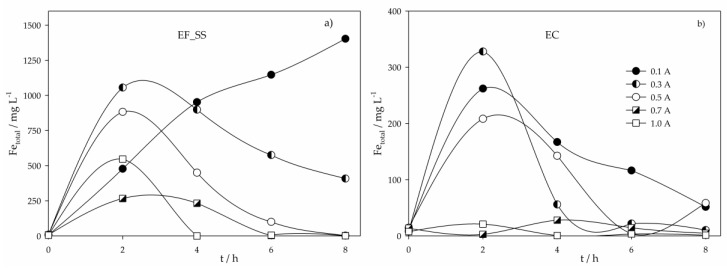
Total dissolved iron variation in time for the assays performed with a SS anode at various applied current intensities and using different electrochemical processes: (**a**) Electro-Fenton; (**b**) Electrocoagulation.

Figure 4 shows the total dissolved iron variation in time for the EF_SS and EC experiments. For EF_SS assays, at 0.1 A, the iron concentration continuously increases. However, for higher I values, Fe concentration increases in the first hours of the assay and then begins to decrease, being the turning point earlier as I increases. Residual Fe concentrations were obtained after 8, 6 and 4 h at the applied current intensities of 0.5, 0.7 and 1.0 A, respectively. Similar behavior was found in EC experiments, but the maximum Fe concentration found was much lower than that registered in EF_SS, and the decrease was observed for all I values. The higher iron increase in EF_SS can be explained by the solution pH, since at acidic conditions, extra dissolution of the anode occurs [25]. The fluctuation observed is mainly due to the iron hydroxide formation, followed by precipitation of the suspended/dissolved matter from the ROC sample, which becomes more pronounced with the increase in applied current intensity.

Energy consumption is a key factor to evaluate an electrochemical treatment. For the electrochemical processes studied at different experimental conditions, the energy consumptions, E, in W·h, were calculated, according to Equation (2), where U is the average cell voltage, in V, determined as the mean value of the cell voltages registered along the 8-h assays, I is the constant applied current intensity, in A, and t is the electrochemical assay duration, 8 h.

E = U · I · t(2)

**Figure 5:**
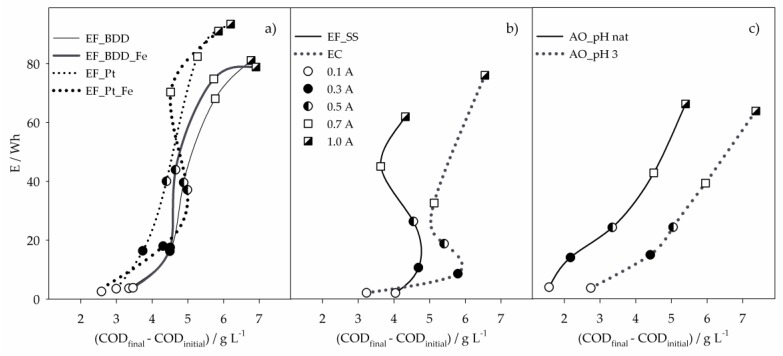
Energy consumptions of the 8-h assays, performed by (**a**) EF with BDD or Pt anodes, (**b**) EF with SS anode and EC, and (**c**) AO, as function of COD removed, for the different applied current intensities. Symbols in (a) and (c) as in (b).

Figure 5 presents the energy consumptions of the 8-h assays as a function of the COD removed, for the different applied current intensities. Results show an increase in E with I, which is due to the increase of cell voltage with I. For the EF experiments performed with BDD and Pt (Figure 5a), it can be seen that, in general, EF_BDD promoted higher COD removals with lower energy consumptions than EF_Pt. Regarding the influence of the presence of extra iron, no significant differences were found in energy consumptions when BDD was used, but a fluctuation was observed with Pt, especially due to the variation in COD removal at some I values, which is explained above.

The EC process presented the lower energy consumptions for I values between 0.1 and 0.7 A (Figure 5b). At 0.3 A, it was the process that removed more COD with the lowest E. However, for I values above 0.3 A, E increased with no gain in COD removal. Despite that, EC showed to be the most efficient process at I values of 0.3 and 0.5 A. Similar behavior was observed for EF_SS (Figure 5b), which, at 0.1 A, presented the highest COD removal with the lowest E, being the most efficient process at this applied current intensity. The trends presented by EC and EF_SS with the increase in I are due to the almost inexistent removal of COD after 2-h assay (Figure 2a).

From all the processes studied, AO was the one that presented a more regular increase of COD removal with I and consequently, E. Energy consumptions obtained for AO_pHnat and AO_pH3 were similar, but AO_pH3 led to much higher COD removals, making this process more efficient than AO_pHnat. In fact, AO_pH3 was the most efficient process of all regarding COD and DOC removals, at I values of 0.7 and 1.0 A.

Considering the results obtained for the parameters analyzed, in the different processes and experimental conditions studied, Table 4 identifies the process that showed the best performance in each parameter, at the different applied current intensities. It can be seen that, for the lowest I, EF_SS led to the highest COD and DOC removals, at the lowest energy consumption. At 0.3 and 0.5 A, EC showed to be the most efficient in COD, DOC and TN removal, presenting the lowest energy consumptions. Also, at 0.7 A, EC presented the highest DOC and TN removals and the lowest E, being the highest COD removal achieved by AO_pH3. At 1.0 A, the lowest energy consumption was obtained with EF_SS, although the highest COD and DOC removals were found for the AO_pH3 process.

Regarding the iron content in the treated samples, AO_pHnat presented, in general, the lowest values, this process also being more suitable for TN removal at 0.1 and 1.0 A.

Although EC and EF_SS are, in general, the processes that present better performance in pollutants removal and energy consumptions, the sludge formed during these treatments can be a drawback in their application. Thus, attending to all the results described, EF using a BDD anode appears as a suitable choice when a low energy consumption process is desired, even with a longer treatment time. The reduction in treatment time can be accomplished by the increase of applied current density, and energy consumption vs. COD removal results (Figure 5) show that, for higher I values, AO processes are the most suitable, especially when the ROC sample has been previously acidified.

## 3. Materials and Methods

### 3.1. Sample Characterization

Reverse osmosis concentrate sample used in this study was collected at a Portuguese intermunicipal sanitary landfill site. The treatment applied at this landfill site comprises two reverse osmosis systems followed by a stripping column. The ROC sample was collected in the reverse osmosis concentrate reservoir.

### 3.2. Electrochemical Experiments

All electrochemical experiments were conducted in batch mode, using an undivided cylindrical glass cell containing 200 mL of the effluent to be treated. In EF and AO experiments, the effluent was continuously stirred at 250 rpm in order to enhance the mass transport of reactants/products toward/from the electrodes. A graphical representation of the experimental setups utilized is shown in Figure S5.

The electrochemical experiments were performed at constant applied current intensities, 0.1, 0.3, 0.5, 0.7, and 1.0 A, using a DC power supply GW, Lab DC, model GPS-3030D (0–30 V, 0–3 A). The initial pH of the samples was adjusted by the addition of concentrated H_2_SO_4_. All chemicals used were of analytical grade and were used without further purification. All the experiments had 8 h duration and were performed at least in duplicate. The values presented for the parameters used to follow the assays are mean values.

#### 3.2.1. Electro-Fenton Experiments

The EF oxidation was conducted using as cathode a carbon-felt piece (Carbone Loraine) with a thickness of 0.5 cm and an immersed area of 110 cm^2^. Three different anodes were tested: a BDD bipolar electrode, with two coated sides, purchased from Neocoat, with an immersed area of 20 cm^2^; a Pt mesh with an immersed area of 2 cm^2^; and a stainless-steel (SS) plate with an immersed area of 20 cm^2^ and thickness of 0.2 cm. The anode was centered in the electrolytic cell and was surrounded by the cathode covering the inner wall of the cell (Figure S5a). Continuous O_2_ saturation at atmospheric pressure was ensured by bubbling compressed air through a fritted glass diffuser at 1 L·min^−1^, starting 10 min before the electrolysis, to reach a steady O_2_ concentration that allowed H_2_O_2_ electrogeneration. To ensure that H_2_O_2_ was being properly produced and was enough to react with the iron, H_2_O_2_ determinations were performed during the assays. All the EF assays were performed at an initial pH of 3.

In the EF assays performed with BDD or Pt as anodes, two initial dissolved iron concentrations were studied: natural iron concentration, 9 ± 5 mg·L^−1^, and 84 ± 14 mg·L^−1^. In the assays with additional iron, iron was electrogenerated in situ, before EF, by performing an electrolysis at a pH of 3, with iron plates as anode and cathode, with an immersed area of 20 cm^2^, at an applied current intensity of 1 A, for 1 minute. At the end of this process, pH was again adjusted to 3 before starting the EF process.

In EF assays performed with the SS anode, sludge was formed, and the samples collected between 2 and 8 h were centrifuged (Meditronic BL-S) before the analytical determinations, to remove the sludge.

#### 3.2.2. Anodic Oxidation Experiments

In the AO experiments, BDD electrode (described above) was used as an anode, and stainless-steel plates, with a similar area to the anode, were used as cathodes. The anode was placed in the center of the electrochemical cell, with two cathodes parallel to the anode, one on each side, with a 0.2 cm gap between each electrode (Figure S5b). AO assays were performed at initial natural pH and at a pH of 3, to compare AO and EF performances at similar initial conditions.

#### 3.2.3. Electrocoagulation Experiments

In the EC assays, both the anode and cathode were stainless steel plates, with a thickness of 0.2 cm and an immersed area of 20 cm^2^, and were placed in parallel with a 1 cm gap between them (Figure S5c). Stainless-steel was chosen as electrodes material since previous assays showed that with this material the iron dissolution was more controlled than with iron plates, without affecting EC performance (data not shown). EC assays were performed at an initial pH of 7, without stirring. Since, during these assays, sludge was formed, the samples collected were centrifuged before the analytical determinations, to remove the sludge.

### 3.3. Analytical Methods

Degradation tests were followed by chemical oxygen demand (COD), total dissolved carbon (TDC), dissolved organic carbon (DOC), dissolved inorganic carbon (DIC), and total dissolved nitrogen (TDN) determinations, which were performed according to the standard procedures [37].

COD determinations were made using the closed reflux titrimetric method [37].

TDC, DOC, DIC, and TN were measured in a Shimadzu TOC-VCPH analyzer combined with a TNM-1 unit. Before TDC, DOC, DIC, and TN determinations, samples were filtered through 0.45 μm membrane filters.

For raw and handled initial samples characterization, ammonium, sulfate, and chloride ions concentrations were determined by ionic chromatography using a Shimadzu 20A Prominence HPLC system that was equipped with a Shimadzu CDD 10Avp conductivity detector. For cations determination, an IC YK-A Shodex (4.6 mm ID×100 mm) column at 40 °C was utilized. The analysis was performed at an isocratic mode with 5.0 mM tartaric acid, 1.0 mM dipicolinic acid and 24 mM boric acid aqueous solution as mobile phase, at a flow rate of 1.0 mL·min^−1^. The anions were quantified using an IC I-524A Shodex (4.6 mm ID ×100 mm) anion column, at 40 °C. The mobile phase was an aqueous solution of 2.5 mM of phthalic acid and 2.3 mM of tris(hydroxymethyl) aminomethane, at a flow rate of 1.5 mL·min^−1^. All solutions for chromatographic analysis were prepared with ultrapure water obtained with Milli-Q^®^ equipment. All chemicals were HPLC grade and supplied by Sigma-Aldrich.

Total dissolved iron and dissolved iron (II) concentrations were determined using a spectrometric method with 1,10-phenanthroline [38]. Since ROC samples absorbed at 511 nm, the absorbance of samples collected during the assays was measured before and after the addition of 1,10-phenanthroline, and the difference between the values was converted into iron concentration.

H_2_O_2_ concentration was determined by the colorimetric metavanadate method [39]. The method is based on the reaction of H_2_O_2_ with ammonium metavanadate in acidic medium, which results in the formation of a red-orange color peroxovanadium complex, with maximum absorbance at 450 nm. Since ROC samples absorbed at 450 nm, the absorbance of samples collected during the assays was measured before and after the addition of metavanate, and the difference between the values was converted into H_2_O_2_ concentration.

For pH and conductivity measurements, a HANNA pH meter (HI 931400) and a Mettler Toledo conductivity meter (SevenEasy S30K) were utilized, respectively.

## 4. Conclusions

Among the electrochemical processes studied, electrocoagulation presented the highest efficiency for the treatment of reverse osmosis concentrate of sanitary landfill leachate. However, sludge formation, with a high content in iron, can hinder its application. The utilization of the electro-Fenton process with the BDD anode also gave interesting results, using natural iron concentration at low current intensities. On the other hand, the electro-Fenton process with the Pt anode showed less efficacy, especially for nitrogen removal. The electro-Fenton process with the SS anode, allowing simultaneous electrocoagulation, led to high COD and DOC removals and to the lowest energy consumptions at the lowest applied current intensity. However, the high amount of iron introduced in the treated effluent and the low nitrogen removal, make this process less appealing.

If treatment time is a key factor, high current densities should be applied and, in this case, anodic oxidation is the best electrochemical choice. By acidifying the effluent, high COD and DOC removals and low energy consumptions are attained. However, the highest nitrogen removals are achieved when anodic oxidation is performed at natural pH conditions.

## Figures and Tables

**Table 1:** Physicochemical characteristics of the reverse osmosis concentrate of sanitary landfill leachate (natural) and of the other samples utilized, with corrections to pH and iron content.

Parameter ^1^	Natural pH	pH = 3	pH = 7	pH = 3 Fe Addition ^2^
COD/g L−1	9.7 ± 0.3	9.5 ± 0.4	9.4 ± 0.5	9.4 ± 0.4
DOC/g·L−1	3.7 ± 0.3	3.3 ± 0.2	3.4 ± 0.3	3.3 ± 0.2
DIC/g·L−1	2.1 ± 0.1	0.10 ± 0.04	1.85 ± 0.09	0.09 ± 0.03
TDN/g·L−1	3.02 ± 0.07	2.91 ± 0.07	3.01 ± 0.03	2.95 ± 0.07
Ammonium/g·L−1	3.58 ± 0.03	3.48 ± 0.01	3.49 ± 0.02	3.58 ± 0.01
Sulfate/g·L−1	2.657 ± 0.006	15.12 ± 0.02	6.06 ± 0.02	14.64 ± 0.02
Chloride/g·L−1	3.53 ± 0.02	3.467 ± 0.006	3.46 ± 0.01	3.53 ± 0.01
Total dissolved iron/mg·L−1	9 ± 5	8 ± 3	11 ± 3	84 ± 14
Dissolved iron (II)/mg·L−1	9 ± 4	8 ± 2	11 ± 3	83 ± 10
pH	8.2 ± 0.1	2.98 ± 0.08	7.1 ± 0.2	2.9 ± 0.2
Conductivity/mS·cm−1	31.3 ± 0.4	35.2 ± 0.4	32.0 ± 0.4	35.5 ± 0.7

^1^ value ± standard deviation. ^2^ iron electrogenerated in situ (1 A; 1 min).

**Table 2:** TN 8-h removals, in mg·L^−1^, for the assays performed at different experimental conditions.

Process	Applied Current Intensity/A				
	0.1	0.3	0.5	0.7	1.0
**EF_BDD**	0	0	284	668	674
**EF_BDD_Fe**	40	313	324	554	531
**EF_Pt**	0	0	0	0	67
**EF_Pt_Fe**	0	70	0	59	295
**EF_SS**	0	91	88	368	349
**EC**	6	434	1011	1017	624
**AO_pHnat**	84	432	680	725	1254
**AO_pH3**	16	221	627	442	770

**Table 3:** Final content in total dissolved iron, in mg·L^−1^, determined for the 8-h assays performed using different electrochemical processes at several applied current intensities.

Process	Applied Current Intensity/A				
	0.1	0.3	0.5	0.7	1.0
**EF_BDD**	62	52	12	36	70
**EF_BDD_Fe**	189	180	113	105	77
**EF_Pt**	8	10	13	15	12
**EF_Pt_Fe**	54	55	48	66	41
**EF_SS**	1403	408	2	2	1
**EC**	52	10	58	5	1
**AO_pHnat**	4	4	5	1	1
**AO_pH3**	15	8	11	4	4

**Table 4:** Performance of the studied electrochemical processes, regarding the different parameters analyzed, at different applied current intensities.

I/a	COD Removal	DOC Removal	TN Removal	Fe Final Content	E
**0.1**	EF_SS	EF_SS	AO_pHnat	AO_pHnat	EF_SS
**0.3**	EC	EC	EC	AO_pHnat	EC
**0.5**	EC	EC	EC	EF_SS	EC
**0.7**	AO_pH3	EC	EC	AO_pHnat	EC
**1.0**	AO_pH3	EF_BDD = AO_pH3	AO_pHnat	EF_SS = EC = AO_pHnat	EF_SS

## Supplementary Materials

The following are available online, Figure S1: COD removal in time for the EF assays performed with a BDD anode, with (a) natural and (b) added iron content, and with a Pt anode, with (c) natural and (d) added iron content, and for the AO assays at (e) natural pH and (f) pH of 3. Figure S2: pH variation in time for: EF assays performed with a BDD anode, with (a) natural and (b) added iron content, with a Pt anode, with (c) natural and (d) added iron content, and with a (e) SS anode; (f) EC assay; AO assays at (g) natural pH and (h) pH of 3. Figure S3: DOC removal in time for: EF assays performed with a BDD anode, with (a) natural and (b) added iron content, with a Pt anode, with (c) natural and (d) added iron content, and with a (e) SS anode; (f) EC assay; AO assays at (g) natural pH and (h) pH of 3. Figure S4: TN removal in time for: EF assays performed with a BDD anode, with (a) natural and (b) added iron content, with a Pt anode, with (c) natural and (d) added iron content, and with a (e) SS anode; (f) EC assay; AO assays at (g) natural pH and (h) pH of 3. Figure S5: Graphical representation of the experimental setups utilized for (a) EF, (b) AO and (c) EC.
