# Supplementary figures and images for: Performance of Electrochemical Processes in the Treatment of Reverse Osmosis Concentrates of Sanitary Landfill Leachate
Source: Molecules. 2019 Aug 10;24(16):2905. doi: 10.3390/molecules24162905 (PMC6720919; doi:10.3390/molecules24162905)

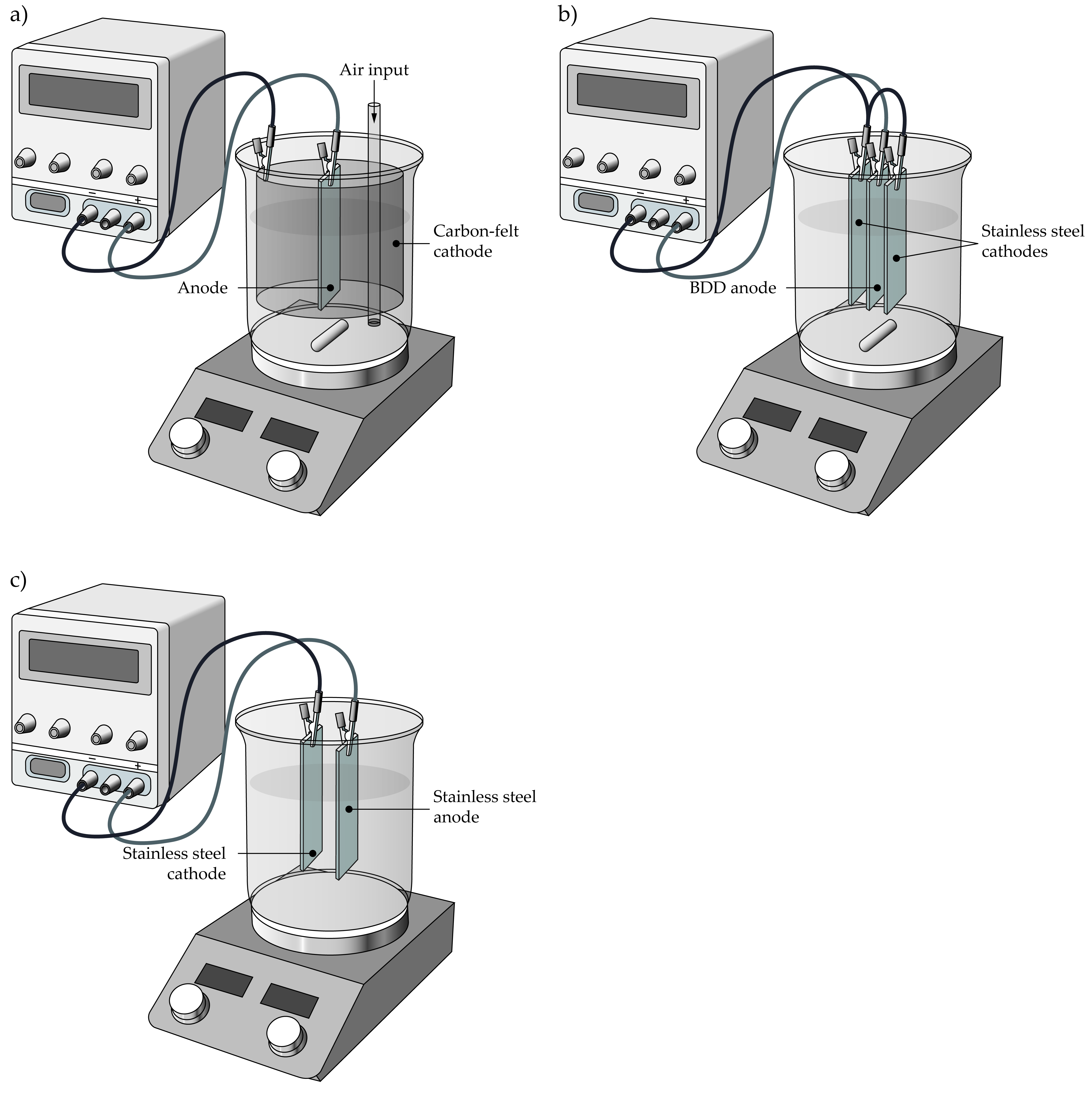

Supplement: Supplementary file 1 [file molecules-24-02905-s001.zip › Fig S5.tif]
